# Impact of gender, organized athletics, and video gaming on driving skills in novice drivers

**DOI:** 10.1371/journal.pone.0190885

**Published:** 2018-01-24

**Authors:** Nancy L. Wayne, Gregory A. Miller

**Affiliations:** 1 Department of Physiology, David Geffen School of Medicine at University of California-Los Angeles, Los Angeles, California, United States of America; 2 Center for the Study of Women, University of California-Los Angeles, Los Angeles, California, United States of America; 3 Westwood Driving School, Los Angeles, California, United States of America; Beihang University, CHINA

## Abstract

Given that novice drivers tend to be young, and teenagers and young adult drivers are involved in the greatest number of accidents, it is important that we understand what factors impact the driving skills of this population of drivers. The primary aim of the present study was to understand the impact of gender, organized athletics, and video gaming on driving skills of novice drivers under real-world driving conditions. Novice driving students having less than five hours driving experience previous to a normal driving lesson were evaluated on their self-confidence (self-reported) prior to the lesson and driving skill evaluated by their instructor during the course of the lesson. Information was collected about gender, age, whether or not the students were involved in organized athletics, and the extent of their video game playing. There was no impact of gender or extent of video game playing on driving skills. Females were significantly less self-confident with driving than males, but this did not translate to gender differences in driving skills. Being involved in organized athletics—either currently or in the past—significantly enhanced driving skills in both females and males. Finally, novice drivers’ age was negatively correlated with driving skills. That is, younger novice drivers (especially males) had better driving skills than older novice drivers. This is counter to popular belief that young drivers lack technical driving skills because they have less experience behind the wheel. Based on the results of the current study, we hypothesize that the relatively high accident rate of younger drivers (especially male drivers) is most likely due to inattention to safety considerations rather than lack of technical driving ability.

## Introduction

Novice drivers tend to be young--teenagers and young adults. The most recent Traffic Safety Facts from the U.S. Department of Transportation [1] reports that young drivers aged 16–20 years are involved in the greatest rate of fatal, injury, and property-damaging accidents of any age group. Not only do these young people lack driving experience compared to most older drivers, but this is an age when the prefrontal cortex of the brain is still maturing. Studies have shown that the prefrontal cortex is responsible for orchestrating high-level cognitive functions that govern goal-directed behaviors or executive functions, attention, and motor coordination [2,3]. There is a relative lack of executive functioning during adolescence and young adulthood due in part to an immature prefrontal cortex [2]. That young drivers are relatively inexperienced and have an immature prefrontal cortex is thought to lead to higher accident rates in this population [4].

Additionally, there are clear gender differences in accident rates, with males having more fatal, injury, and property-damaging accidents than females at every age reported (16 to > 74 years). This gender difference is especially pronounced at the youngest age range of 16–20 years [1].

While it is not yet possible to speed-up aging and maturation of the brain in teenagers or young adults, it may be useful to understand the major factors that influence driving skills of novice drivers in real-world driving conditions.

Few studies have investigated the influence of gender or sports on driving skills of novice drivers. Work that investigated driving skills through simulation testing showed no impact of gender; but, there was a positive influence of high-level sport competition on one aspect of driving—time to brake [5]. Work that investigated driving skills on the road showed that team sports players were better at a peripheral detection task than drivers who did not play team sports [6]. As far as we know, there are no published studies investigating the influence of home video gaming on driving skills.

The aim of the present work was to understand the impact of gender, organized athletics, and video gaming on driving skills of novice drivers in real-world driving conditions. According to the Federal Highway Administration’s Office of Operations [7], the Westside of Los Angeles where this study was conducted, and where most of the subjects live, work, and/or go to school, has one of the worst physical traffic bottlenecks in the U.S. According to the California Office of Traffic Safety [8], the City of Los Angeles ranks third of fourteen California cities with a population over 250,000 in terms of fatal and injury vehicle accidents (behind #1 Anaheim and #2 Santa Ana). That makes learning and practicing to drive in this area difficult, frustrating and dangerous—a challenging environment, especially for novice drivers.

## Methods

### Experimental design

This study was conducted in real-world, real-time conditions during the course of regular driving instruction on the Westside of Los Angeles, comprising the area from La Brea Blvd. on the East to the Pacific Ocean on the West, and from Malibu on the North to Playa Vista on the South.

Novice drivers were students at the Westwood Driving School in Los Angeles. They were selected as potential subjects for this study based on having less than five hours driving experience (self-reported) previous to the driving lesson when the study was conducted. One hundred novice drivers were recruited for the study, 50 females and 50 males. The 100 subjects were instructed and evaluated by the same driving instructor (G. Miller) who is also an author of this paper. Because of inter-instructor variability in assessing driving skills of the students, a single driving instructor was recruited to perform all skill evaluations during the course of the normal driving lesson.

Before beginning to drive at the time of the first lesson, subjects were asked in writing, “How confident are you about your driving skills? Circle one: 1 2 3 4.” The driving instructor verbally stated that, "1 is like 'uh-oh, this is going to be a disaster’ and 4 is like 'I've got this, no problem'." They then rated their level of confidence. The instructor was not privy to the subject's self-confidence rating until after the end of the lesson.

During a two-hour driving lesson involving car control and maneuvers in traffic on commercial and residential streets, the instructor rated the subject's skills on a scale of 1–4, the rating system used by Westwood Driving School. A rating of 1 means the student requires significantly more instruction and practice; 2 means the student requires some instruction and more practice; 3 means the student requires no more instruction but could use more practice; and, 4 means the student's driving abilities are sufficient to pass the California Department of Motor Vehicle (DMV) behind-the-wheel driving test.

At the end of the lesson, the instructor verbally notified the subject that the present study was being conducted, explained what the purpose of the study was, and asked if they would be willing to participate. If they agreed, they signed and were offered a copy of an Informed Consent form. They were given the right to withdraw their consent at any point during the study. There were no withdrawals. After signing the consent form, subjects were asked two follow-up questions by the instructor: A) Are they currently, or have they previously, participated in any sports or organized athletics, and if so, which (see Table 1 for the different organized athletic activities that subjects were involved in either currently or in the past); and, B) Do they currently, or have they previously, played video games, “Not at all” (0), “A little” (1), “A lot” (2), or “All the time” (3). There are typically four back-to-back two-hour lessons each day. Because the study was embedded in real-world driving lessons with a tight time schedule, there was very little time to ask questions of the subjects.

**Table 1:** Organized athletic activities (team sports and non-team athletics) that subjects were involved in either currently or in the past.

**Team Sports**	badminton, baseball, basketball, football, field hockey, ice hockey, soccer, softball, tennis, volleyball, water polo
**Non-Team Athletics**	boxing, color guard, dance, fencing, golf, judo, ju-jitsu, karate, ping pong, skating, skateboarding, surfing, swimming, track, yoga

### Ethics statement

The study and all methods were carried out in accordance with and approved by the institutional review board of UCLA (protocol #16–001436; approval period from November 4, 2016 through November 3, 2017).

### Statistical analysis

Data are shown as the mean ± SEM. Data in each comparison group were analyzed for normal distribution using the D’Agostino-Pearson omnibus normality test. Because female and male age, female driving skills rating, and female video game rating were not normally distributed, we used non-parametric statistical analyses. For analysis of the impact of athletic activities on driving skills in which there were more than two groups, statistical significance between was determined by two-tailed, Kruskal-Wallis test. For comparisons of variables between two groups, statistical significance was determined by two-tailed, Mann-Whitney test. For correlations of two variables, statistical significance was determined by two-tailed, Spearman Correlation Test. Statistical analysis used GraphPad Prism 7 (GraphPad Software, Inc., La Jolla, CA). Values were considered significantly different if *p* < 0.05.

## Results

Table 2 shows that there were no significant gender differences in age or hours behind the wheel prior to the first driving lesson for novice drivers. Therefore, those two variables would not have a significant impact on any gender differences observed in this study.

**Table 2:** Age, hours behind the wheel prior to first driving lesson, self-confidence rating, driving skill rating, and video game rating of female and male subjects (mean ± SEM).

	FEMALE	MALE	P-value
**Age (years)**	18.2 ± 2.6; range: 15.5–29 years	17.9 ± 2.6; range: 15.5–25 years	P > 0.05
**Hours behind wheel**	1.5 ± 0.2	1.4 ± 0.2	P > 0.05
**Self-confidence rating**	1.9 ± 0.3	2.4 ± 0.1	P = 0.01
**Driving skill rating**	2.4 ± 0.34	2.5 ± 0.09	P > 0.05
**Video game rating**	0.36 ± 0.05	1.38 ± 1.0	P < 0.0001

**Fig 1:**
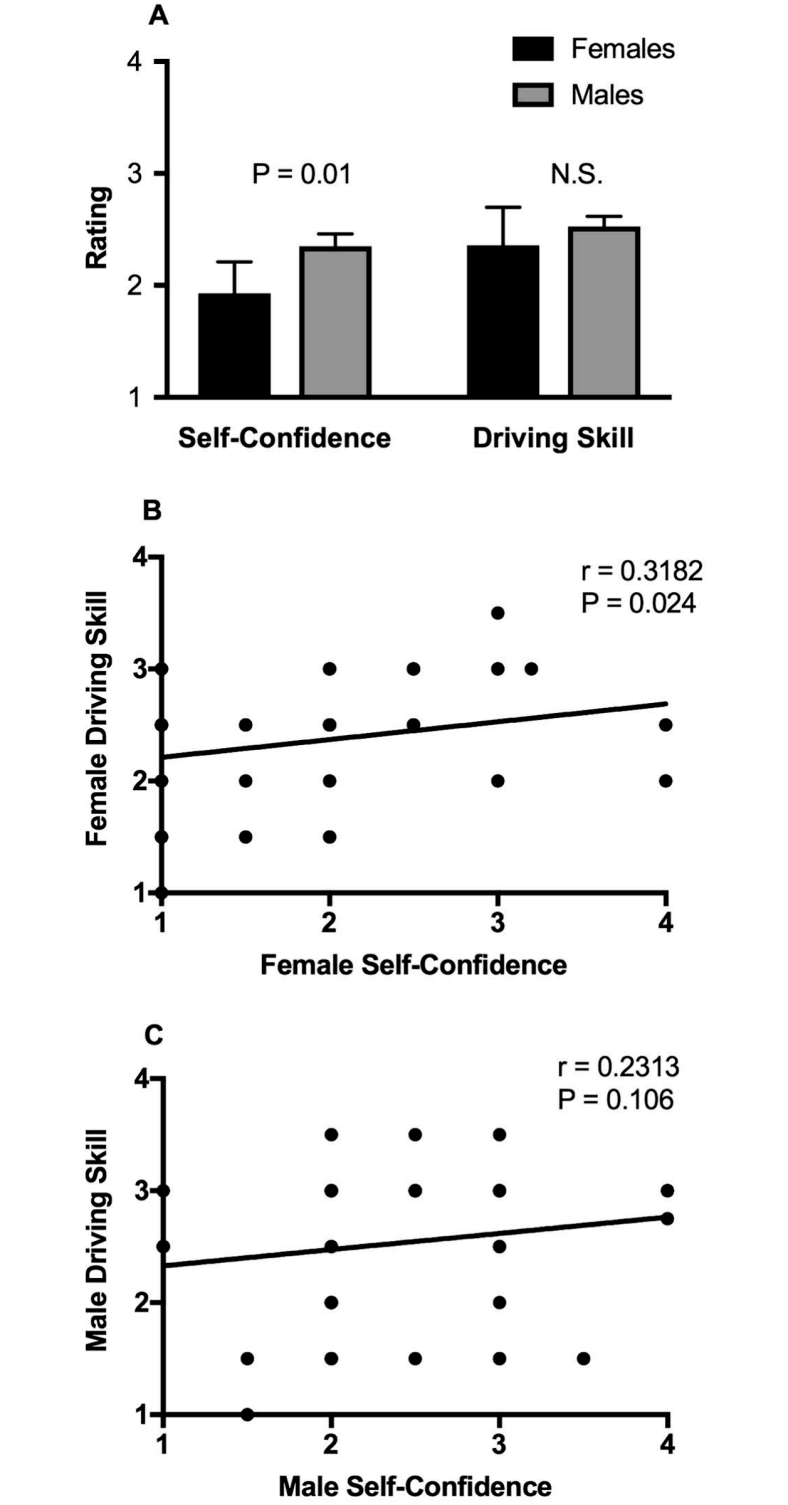
Impact of gender on self-confidence rating and driving skill rating. (A) Comparison of self-confidence rating (left) and driving skill rating (right) between males and females. N.S. means no significant difference in driving skill rating between genders. (B) Correlation between self-confidence rating and driving skill rating for females. (C) Correlation between self-confidence rating and driving skill rating for males. Trend lines of the linear regression analysis are shown in panels B and C. There are fewer than 50 visible data points in panels B and C because x-y coordinates of many values were identical and therefore overlapping. r refers to the correlation coefficient.

Fig 1 and Table 2 show that females were significantly less self-confident in their driving skills than males (Fig 1A), and yet there was no gender difference in driving skill rating at the end of the lesson (Fig 1A). There was a significant correlation between self-confidence rating and driving skill rating for females (Fig 1B), but not for males (Fig 1C). These findings suggest that the association between self-confidence and driving skill ratings in females does not translate to a gender difference in driving skills.

**Fig 2:**
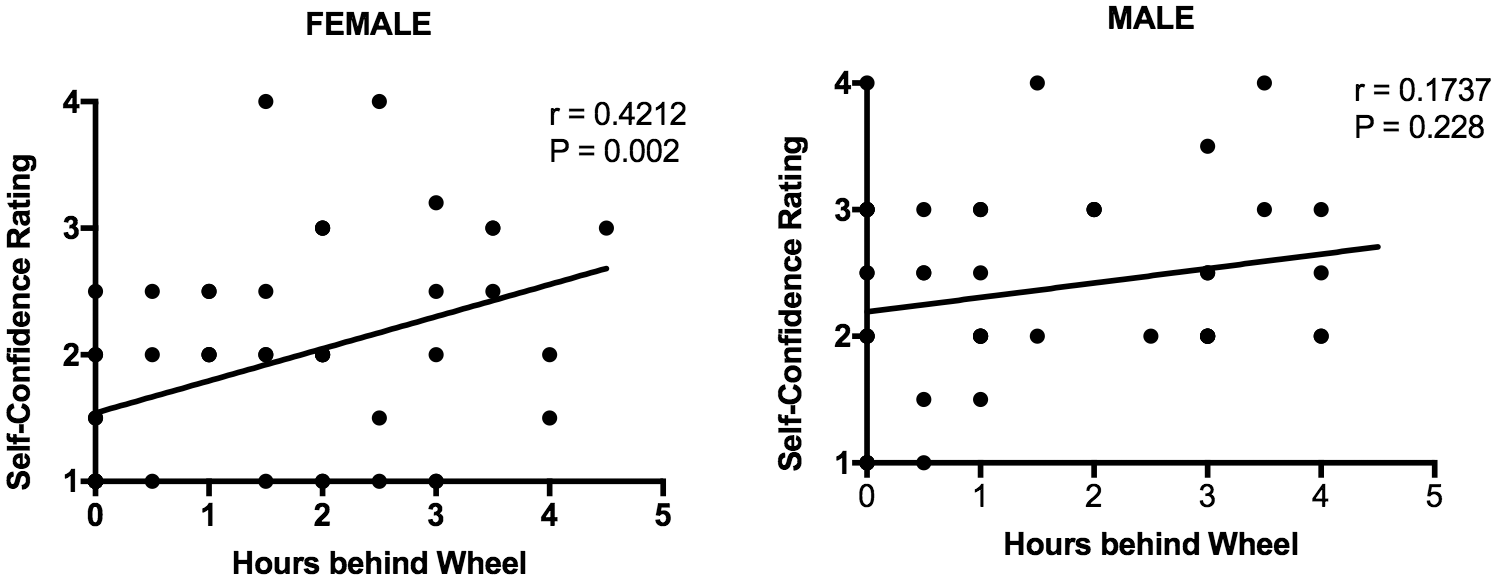
Correlation between hours behind the wheel prior to first lesson and self-confidence rating. Left: females; Right: males. Trend lines of the linear regression analysis are shown in left and right panels. There are fewer than 50 visible data points in panels B and C because x-y coordinates of many values were identical and therefore overlapping. r refers to the correlation coefficient.

Fig 2 shows a significant correlation between hours behind wheel and self-confidence for females—the more hours behind the wheel prior to first lesson, the greater their self-confidence. This was not the case with males where there was no correlation. However, there was no correlation between hours behind the wheel prior to first driving lesson (< 5 hours) and driving skill reported by instructor for females (r = 0.142, P = 0.326) or males (r = -0.052, P = 0.720) (data not shown).

**Fig 3:**
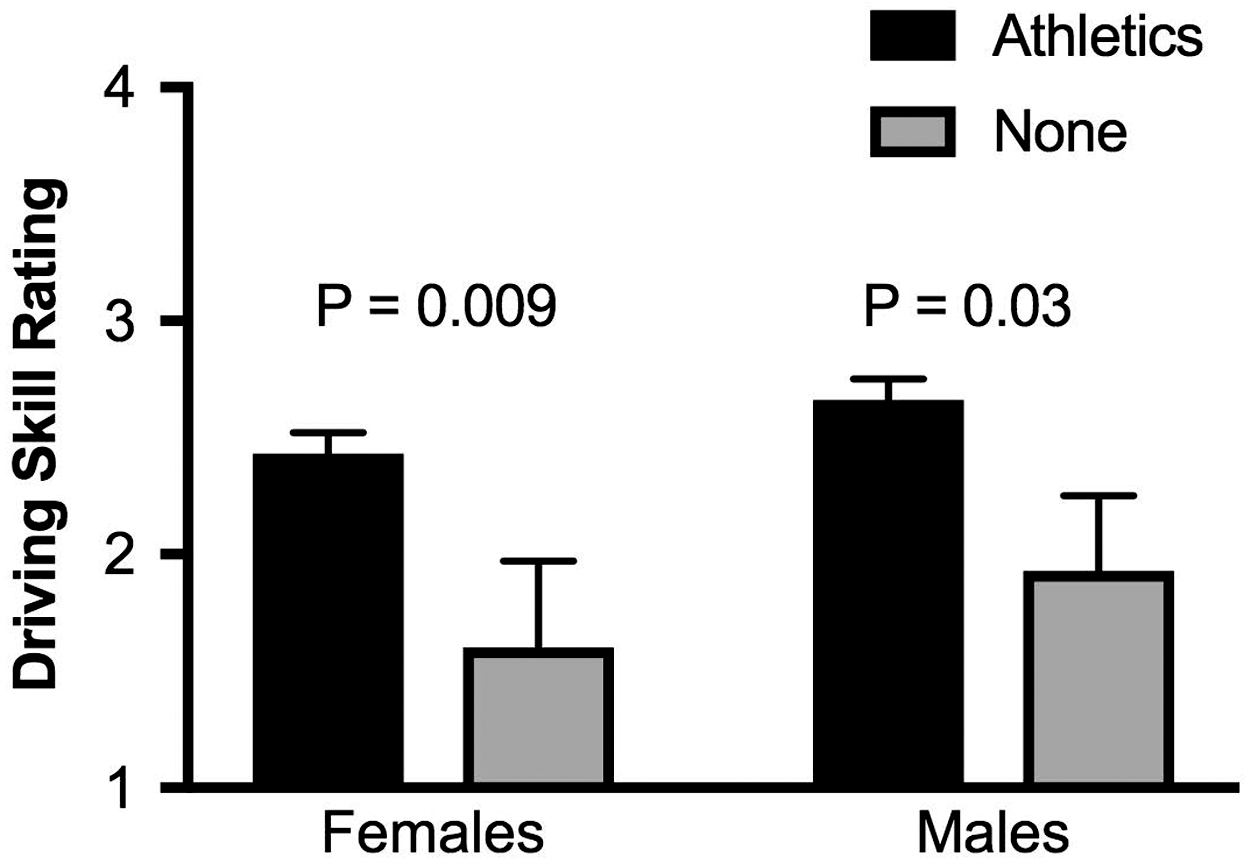
Impact of involvement in organized athletics on driving skill rating. Left: females; Right: males. There was no significant difference between team sports, non-team athletic activity, and past athletic activity, so that data was combined into a single category of organized athletics (Athletics: female, n = 45; male, n = 43) and compared to subjects reporting no involvement in organized athletics (None: female, n = 5; male, n = 7).

A striking finding was that the vast majority of novice drivers in our study were involved in organized athletic activities, either at the time of the study or in the past: 90% of females (45 out of 50) and 86% of males (42 out of 50). For both females and males, there were no significant differences in driving skill if athletic activity was a current team sport, a current non-team sport, or an organized athletic activity practiced in the past. Therefore, we combined the data from those three groups and compared them with drivers who reported no athletic activities in Fig 3. Both female and male novice drivers who reported no current or past athletic activity showed significantly lower driving skills than those who were involved in athletic activities. During the lesson and when a driving skill rating was assigned at the end of the lesson, the instructor was blind to whether or not subjects were involved in athletic activities, however we cannot rule out implicit subjective bias on the part of the instructor, or explicit indications (sports clothing) on the part of the subject that might have unconsciously influenced the instructor’s evaluation.

**Fig 4:**
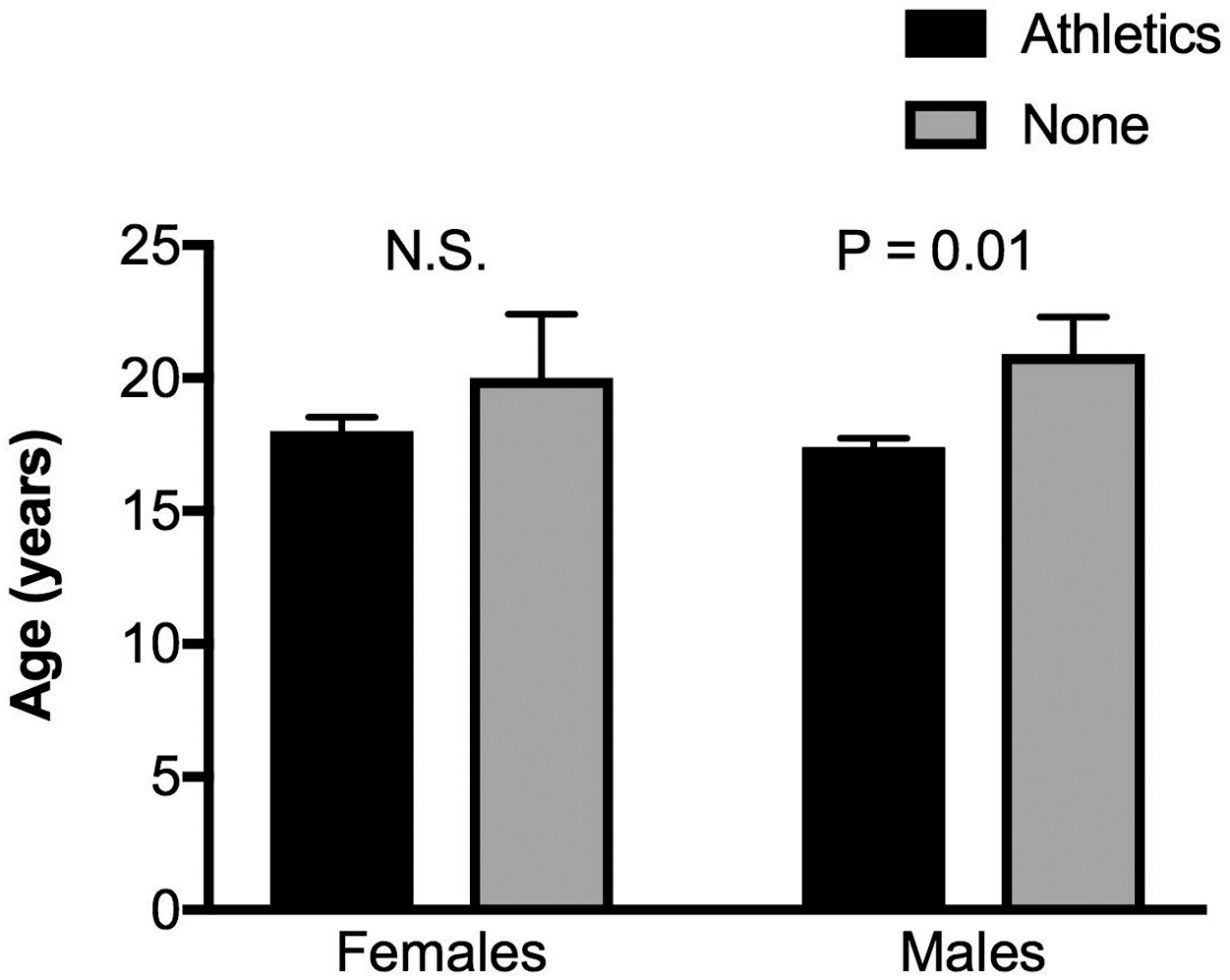
Relationship between age, gender, and involvement in organized athletics. Left: females; Right: males. N.S. means no significant difference in age between athletic and non-athletic (None) in females.

**Fig 5:**
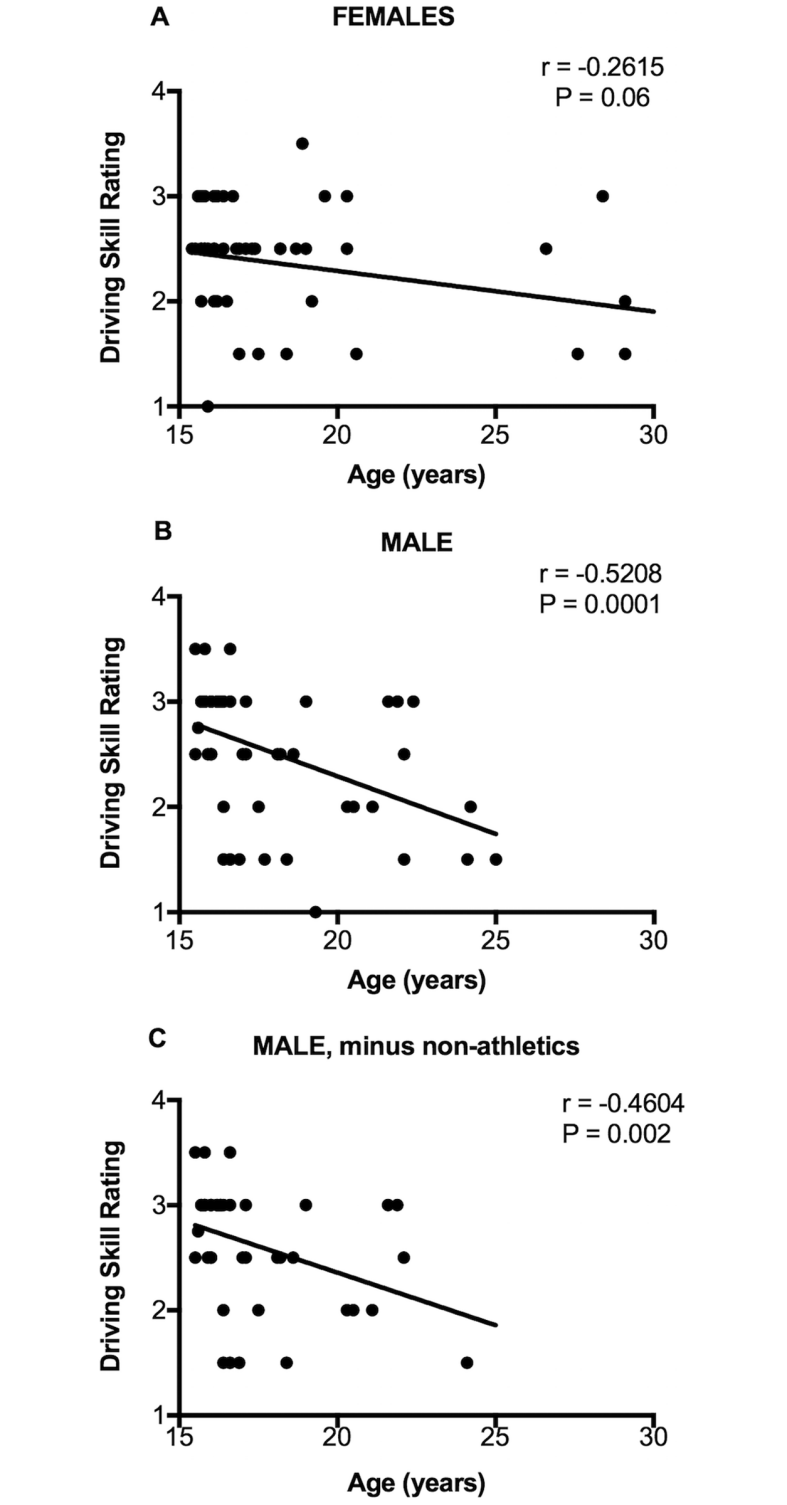
Correlation between age and driving skill rating. (A) Females; (B) All males; (C) Males minus those who were non-athletic. Trend lines of the linear regression analysis are shown in all panels. There are fewer than 50 visible data points because x-y coordinates of many values were identical and therefore overlapping. r refers to the correlation coefficient.

Fig 4 shows that there was no age difference between females who were involved in athletics versus those who were not involved in athletics. However, that was not the case with males. Males who were involved in athletics were significantly younger than those who were not ever involved in organized athletics. Fig 5 shows that there was a negative correlation between age and driving skills for all males (panel B) and for males minus those who were non-athletic (panel C); that is, the older the novice male driver the lower his driving skill rating regardless of athletic activity. Although there was a tendency for older novice females to also have lower driving skill ratings, this negative correlation didn’t reach statistical significance (panel A). We have no plausible explanation for why the older novice drivers have worse driving skills.

**Fig 6:**
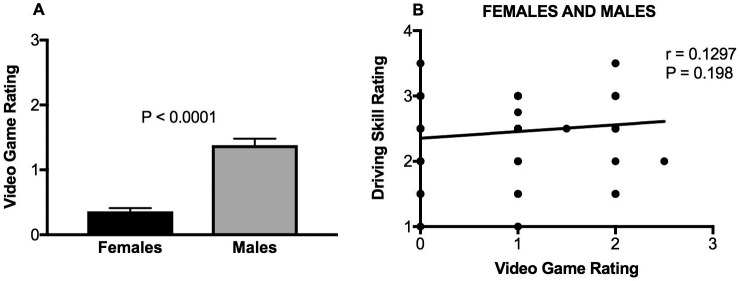
Relationship between playing video games, gender, and driving skills. (A) Gender differences in video game rating; (B) Correlation between video game rating and driving skill rating for all 100 subjects. Trend line of the linear regression analysis is shown in panel B. There are fewer than 100 visible data points because x-y coordinates of many values were identical and therefore overlapping. r refers to the correlation coefficient.

Fig 6 and Table 2 show that video game rating was significantly lower for females than males (Fig 6A), meaning that females play less video games in general. We expected to find that playing video games would increase a driver’s skill, but an analysis of all 100 subjects showed no correlation between video game rating and driving skills (Fig 6B). The same held true when female and males were analyzed separately (data not shown). Importantly, we have no information on what types of video games the subjects were playing.

## Discussion

The present investigation is unique in that it: 1) focuses on a group of brand-new drivers with less than five hours experience prior to the lesson during which they were evaluated; 2) takes place under real-world driving conditions in which traffic and the behavior of other drivers is unpredictable; 3) studies drivers who were not aware they were being evaluated during the lesson as they would be in a simulated driving test; and 4) measures the impact of self-confidence, gender, organized athletics, and video gaming on driving skills in novice drivers. While there was a robust positive impact of organized athletics on driving skills, gender had little to no impact, and playing video games had no impact whatsoever. Because this study took place under the real-world conditions of regular driving school lessons, there are certain caveats and restrictions that could impact generalizing interpretations of the data: a single instructor evaluating driving skills; a single driving school; and, restriction to a local area of Los Angeles. Although this design was important for quality control purposes, it’s important to keep these restrictions in mind when thinking about potential broad implications of the study.

### No gender difference in driving skills

One of the main outcomes from this study is that novice females and males had similar driving skill ratings. This is supported by earlier work in simulated driving tests showing no gender differences in skill level [5]. However, the present study also showed that females were initially less confident in their driving skills than males, and their confidence was associated with how well they drove—the more confident, the better the driving skill rating. This was not the case for males in which self-confidence was not correlated with skill level. So, although there was no overall gender difference in driving skill rating, there was an association of self-confidence with driving skills in females only. It is also worth noting that female drivers’ confidence was positively correlated with hours behind the wheel prior to the lesson—the more hours behind the wheel, the more self-confident the female driver.

The issue of relative lack of confidence in females and how it might impact performance has been an area of investigation in the STEM fields of science, technology, engineering, and math. Studies have shown that females tend to underestimate their exam test scores relative to males, while males tend to overestimate their test scores relative to females in the areas of math and chemistry [9,10]. For the first time, the present study shows that the confidence-competence conundrum for females extends to the realm of driving. In this case, females are less confident in their driving abilities than males, but have equal driving skill rating.

The present study showed no difference in driving skill between male and female drivers with under 5 hours’ experience behind the wheel, however accident data patterns in the U.S. show that males get into more accidents than females [1]. This suggests there are at least two different kinds of driving skills: technical and safety. One study using self-assessments and questionnaires of undergraduate students in Turkey investigated the influence of gender on two types of driving skills: safety and perceptual-motor [11]. Males scored higher on perceptual-motor skills but reported greater car accidents than females. On the other hand, females scored higher in safety skills and reported lower car accidents than males. The gender difference in accidents was impressive: females had 59% less total accidents, 54% less active accidents, and 67% less passive accidents than male drivers. Although this study was performed in Turkey, it suggests that stereotypical male machismo attitudes and behaviors that are prevalent across nationalities and cultures are a leading indicator of accident probability—not technical driving ability *per se*.

### A positive correlation between athletics and driving skills

A second main outcome of this study is that practicing organized athletics of any kind, solo or team sports, either past or present, is associated with enhanced driving skills in both females and males. Previous work has investigated the impact of high-level team sports activities on driving skills. In one study [5], the authors hypothesized that improved spatiotemporal functions acquired during elite team sports practice would transfer to competency in driving. In that study, “athletic” subjects were defined as individuals on a Division I university athletic team; while “non-athletes” were not on an elite sports team. Simulated driving assessed two highly specific aspects of driving: Preferred time headway (PTH) trial participants drove behind a lead vehicle and were instructed to maintain a constant distance for approximately 5 min; 2) Braking trial participants drove behind a lead vehicle that braked. There was no effect of athletics on PTH, but there was an enhanced response in the braking test. Based on the results of the current study, the inconsistent effects of athletics on driving performance shown in the simulation study by Hancock and colleagues [5] may be due to the low number of subjects (24 total), and that the ‘non-athlete’ control group might have included subjects who were involved in non-elite athletic activity during or prior to the study.

In a second study [6], subjects were divided into “team sports players” with at least three years of sports practice and “non-team sports players”. They were given a very specific Peripheral Detection Task during driving on the road, and team sports players scored more highly than non-team sports players. The authors noted that people who play sports have better peripheral vision compared to those not engaged in sports, and that peripheral vision is an important aspect of driving safety. The present study indicates that involvement in elite team sports is not required for people to acquire the necessary spatiotemporal competency that translates to better driving skills—any organized athletic activity will do.

### No correlation between playing video games and driving skills

The present study found no correlation between playing video games and driving skill in either males or females. This was a surprising outcome because other studies have shown that playing action video games improves spatial cognition [12,13]. Previous work studying older adults [14,15] also found that spatial acuity is an important element in driving competency. Male subjects in the current study reported playing video games nearly four times more than females, which is of sociological and cultural interest, but did not translate to any significant gender difference in driving skill rating.

### Younger novice drivers exhibit higher driving skill ratings than older novice drivers

Another surprising result of the present study was that younger male drivers had a higher skill rating than older males, even after non-athletic (lower-skilled) subjects were removed from the dataset. This counter-intuitive trend was also present in the female drivers but did not achieve the same level of statistical significance.

The present study was performed in novice drivers whose average age was 18 years (both females and males). In California, as elsewhere in the U.S., it is a requirement that anyone under the age of 18 must pay a fee to take formal driver’s education classes and a minimum of six hours of on-road driving instruction from a certified driver’s education program. In California, they must also wait six months after receiving a learner’s permit before they are permitted to take the DMV Driver’s Road Test, making the licensing process for younger drivers more expensive and time-consuming. But is this age-mandated driver-training effective in reducing accidents compared to novice drivers who are older than 18 years and don’t require the training? The present study showed a negative correlation between age and driving skills of novice drivers, which was most pronounced in males indicating that in fact it is older males who might require more training or different training. Further, according to the State of California DMV, Section 5 on Driver Training and Teenage Drivers [16]:

In 1965, [California DMV] R&D performed a statistical evaluation (Research Report No. 21) of the driving records of thousands of teenage drivers. This study found no evidence that driver training was associated with reduced accidents. Further, it found no evidence to support raising the licensing age to 18 for applicants who had not completed driver training. To our knowledge, this was the first empirical effectiveness study questioning the alleged value of driver training. Despite the findings and recommendations of the study, the legislature passed a 1967 law (still in force) requiring persons to complete an approved driver training course in order to be licensed prior to 18. This study is obviously not an example of successful policy influence but it is cited for historical context and consistency with later studies showing behind-the-wheel training to be of little or no value in decreasing the accident rate of young drivers.

Given these findings, perhaps the California DMV—and other states’ Graduated Driver's License programs—should consider ending age-based mandatory driver’s education, and focus more on safety training for all novice drivers of any age [17–19].

A study based on data from New Jersey Traffic Safety Outcomes data warehouse [19] found that older novice drivers were involved in less accidents than younger novice drivers in the short term (within 3 months of obtaining their license), but more accidents in the mid-term (within 3 years of obtaining their license). This supports the theory that the higher rate of accidents among younger novice drivers is potentially due solely to behavioral factors, not driving ability per se. Further study would be required to determine if, within those populations, drivers who practice organized athletics have a different accident rate than non-athletes, and therefore form differentiable risk-groups.
